# Correction: Moderate aerobic exercise, but not anticipation of exercise, improves cognitive control

**DOI:** 10.1371/journal.pone.0308314

**Published:** 2024-07-31

**Authors:** Maximilian Bergelt, Vanessa Fung Yuan, Richard O’Brien, Wellington Martins dos Santos, Laura E. Middleton

The authors are listed out of order. Please view the correct author order, affiliations, and citation here:

Maximilian Bergelt^1^, Vanessa Fung Yuan^1,2^, Richard O’Brien^1^, Wellington Martins dos Santos^3^, Laura E. Middleton^1^

**1** Department of Kinesiology, University of Waterloo, Waterloo, ON, Canada, **2** Department of Kinesiology, Brock University, St. Catharines, ON, Canada, **3** Department of Physical Education, University of Campinas, Campinas, São Paulo, Brazil

Bergelt M, Fung Yuan V, O’Brien R, Martins dos Santos W, Middleton LE (2020) Moderate aerobic exercise, but not anticipation of exercise, improves cognitive control. PLOS ONE 15(11): e0242270. https://doi.org/10.1371/journal.pone.0242270
