# Supplementary material for: Moderate aerobic exercise, but not anticipation of exercise, improves cognitive control
Source: PLoS One. 2020 Nov 13;15(11):e0242270. doi: 10.1371/journal.pone.0242270 (PMC7665798; doi:10.1371/journal.pone.0242270)
Supplement: S1 File — (PDF) [file pone.0242270.s001.pdf]

Anticipation and Exercise Summary Analysis

Max Bergelt

6/26/2020

```
## ‘summarise()‘ regrouping output by ’participant’ (override with ‘.groups‘ argument)
```

	. (N = 31)
**gender**	
1	14 (45)
2	17 (55)
**age**	
minimum	20.00
median (IQR)	22.00 (22.00, 22.00)
mean (sd)	22.00 ± 0.90
maximum	25.00
Unknown	8/31 (26)
**ipaq**	
minimum	360.00
median (IQR)	2,278.00 (1,247.25, 3,972.25)
mean (sd)	2,797.33 ± 1,897.73
maximum	6,557.50
Unknown	16/31 (52)

EXERCISE DATA DESCRIPTION

	condition: 1 (N = 310)	condition: 2 (N = 310)	condition: 3 (N = 310)
**hr**			
minimum	50.00	53.00	81.00
median (IQR)	74.00 (68.00, 82.00)	76.00 (69.00, 83.00)	130.00 (116.00, 145.00)
mean (sd)	75.09 ± 9.97	76.62 ± 10.23	130.89 ± 19.92
maximum	102.00	109.00	180.00
Unknown	20/310 (6)	40/310 (13)	31/310 (10)
**watts**			
minimum	0.00	0.00	0.00
median (IQR)	0.00 (0.00, 0.00)	0.00 (0.00, 0.00)	60.00 (45.00, 90.00)
mean (sd)	0.00 ± 0.00	0.00 ± 0.00	66.47 ± 28.76
maximum	0.00	0.00	125.00
Unknown	20/310 (6)	40/310 (13)	20/310 (6)
**rpe**			
minimum	Inf	Inf	7.00
median (IQR)	NA (NA, NA)	NA (NA, NA)	13.00 (13.00, 13.00)
mean (sd)	NaN ± NA	NaN ± NA	13.15 ± 1.01
maximum	-Inf	-Inf	16.00
Unknown	310/310 (100)	310/310 (100)	20/310 (6)
**rpm**			
minimum	0.00	0.00	83.00
median (IQR)	0.00 (0.00, 0.00)	0.00 (0.00, 0.00)	90.00 (88.00, 91.00)
mean (sd)	0.00 ± 0.00	0.00 ± 0.00	89.51 ± 2.53
maximum	0.00	0.00	97.00
Unknown	20/310 (6)	40/310 (13)	20/310 (6)

HEART RATE BY CONDITION AND TIME

% HR Max

Time Point	Rest	Deception	Exercise	Significance
Pre-Reveal	39.28 (5.77)	40.91 (5.79)	40.67 (6.23)	0.289
Pre-Intervention	39.39 (4.79)	40.08 (5.85)	39.31 (5.7)	0.78
During Intervention	37.91 (4.62)	38.6 (4.77)	66.91 (10.1)	<.001
Post-Intervention	38.19 (4.53)	38.72 (5.84)	46.13 (8.79)	<.001

Raw HR

Time Point	Rest	Deception	Exercise	Significance
Pre-Reveal	77.76 (11.35)	81 (11.46)	80.52 (12.31)	0.287
Pre-Intervention	78 (9.51)	79.33 (11.48)	77.83 (11.27)	0.784
During Intervention	75.05 (9.15)	76.43 (9.44)	132.48 (20.13)	<.001
Post-Intervention	75.62 (8.97)	76.67 (11.55)	91.34 (17.43)	<.001

	. (N = 558)
**LISAS.score**	
minimum	236.04
median (IQR)	346.05 (316.85, 381.22)
mean (sd)	352.94 ± 49.21
maximum	539.39
Unknown	52/558 (9)
**meanRT**	
minimum	214.12
median (IQR)	335.32 (307.35, 369.42)
mean (sd)	341.66 ± 49.15
maximum	539.39
Unknown	52/558 (9)
**proportion.error**	
minimum	0.00
median (IQR)	0.02 (0.00, 0.06)
mean (sd)	0.05 ± 0.06
maximum	0.34
Unknown	52/558 (9)

	Sum Sq	Mean Sq	NumDF	DenDF	F value	Pr(>F)
session	6202.91120	3101.45560	2	425.02716	5.4681495	0.0045212
condition	4291.00557	2145.50278	2	423.87582	3.7827174	0.0235329
timePoint	28420.92943	14210.46471	2	419.90511	25.0543474	0.0000000
congruency	128263.60636	128263.60636	1	419.81855	226.1404550	0.0000000
gender	876.23749	876.23749	1	27.86115	1.5448867	0.2242508
noRespRate	32321.07510	32321.07510	1	433.92226	56.9850080	0.0000000
session:condition	4450.71760	1112.67940	4	436.53286	1.9617554	0.0993791
session:timePoint	7086.88231	1771.72058	4	419.88627	3.1237052	0.0149830
condition:timePoint	9378.21171	2344.55293	4	419.86085	4.1336610	0.0026944
session:congruency	167.33588	83.66794	2	419.81438	0.1475142	0.8628949
condition:congruency	63.21945	31.60973	2	419.81832	0.0557308	0.9458007
timePoint:congruency	26.17619	13.08810	2	419.81403	0.0230755	0.9771899
session:condition:timePoint	3212.35937	401.54492	8	419.85638	0.7079604	0.6845988
session:condition:congruency	1701.48874	425.37218	4	419.81827	0.7499700	0.5584414
session:timePoint:congruency	513.41710	128.35428	4	419.81504	0.2263003	0.9236441
condition:timePoint:congruency	298.03500	74.50875	4	419.81430	0.1313657	0.9708822
session:condition:timePoint:congruency	588.62662	73.57833	8	419.81379	0.1297253	0.9979554

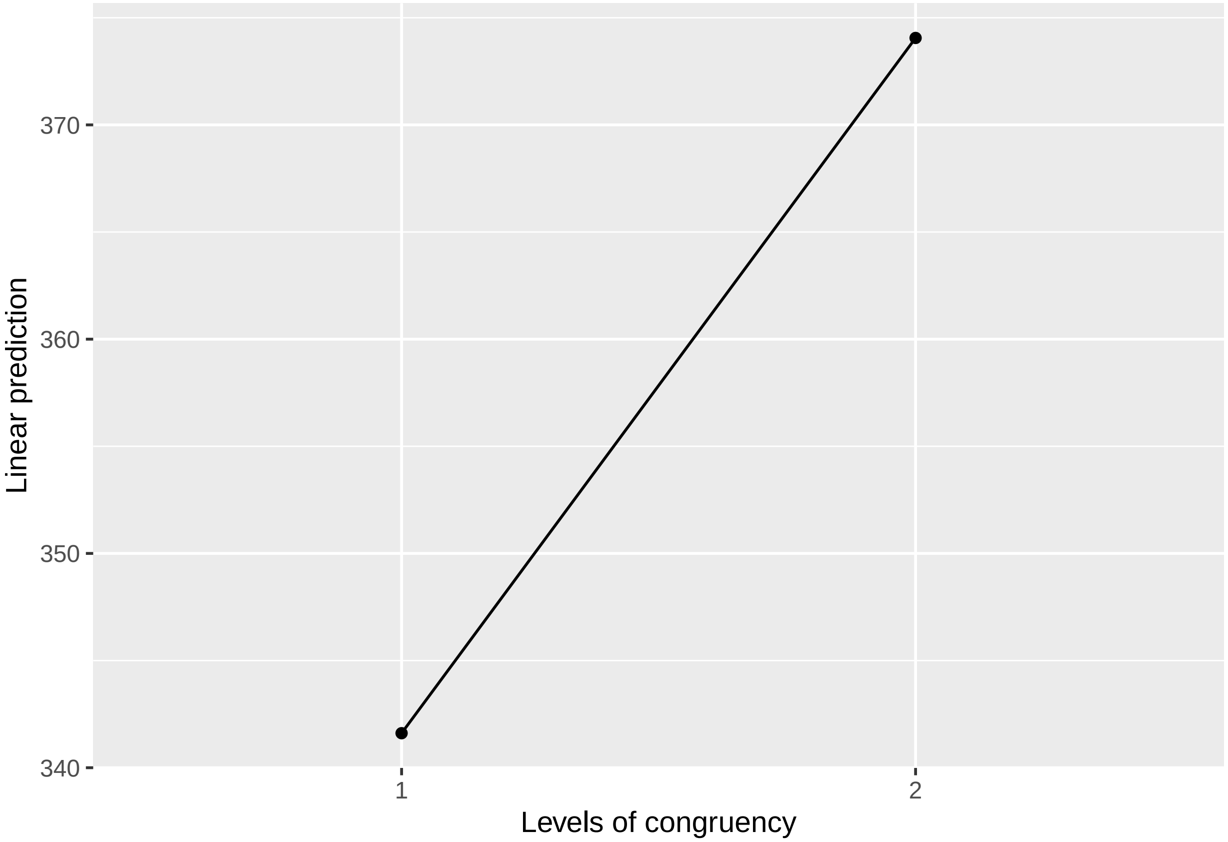


```
## $emmeans
##  congruency emmean   SE   df lower.CL upper.CL
##  1             342 8.07 28.8      323      361
##  2             374 8.07 28.8      355      393
##
## Results are averaged over the levels of: session, condition, timePoint, gender
## Degrees-of-freedom method: satterthwaite
## Confidence level used: 0.95
## Conf-level adjustment: sidak method for 2 estimates
##
## $contrasts
##  contrast estimate   SE  df t.ratio p.value
##  1 - 2       -32.4 2.16 420 -15.038 <.0001
##
## Results are averaged over the levels of: session, condition, timePoint, gender
## Degrees-of-freedom method: satterthwaite
```

```
##  contrast effect.size    SE  df lower.CL upper.CL
##  (1 - 2)        -1.36 0.101 420    -1.56    -1.16
##
## Results are averaged over the levels of: session, condition, timePoint, gender
## sigma used for effect sizes: 23.82
## Degrees-of-freedom method: inherited from satterthwaite when re-gridding
## Confidence level used: 0.95
```

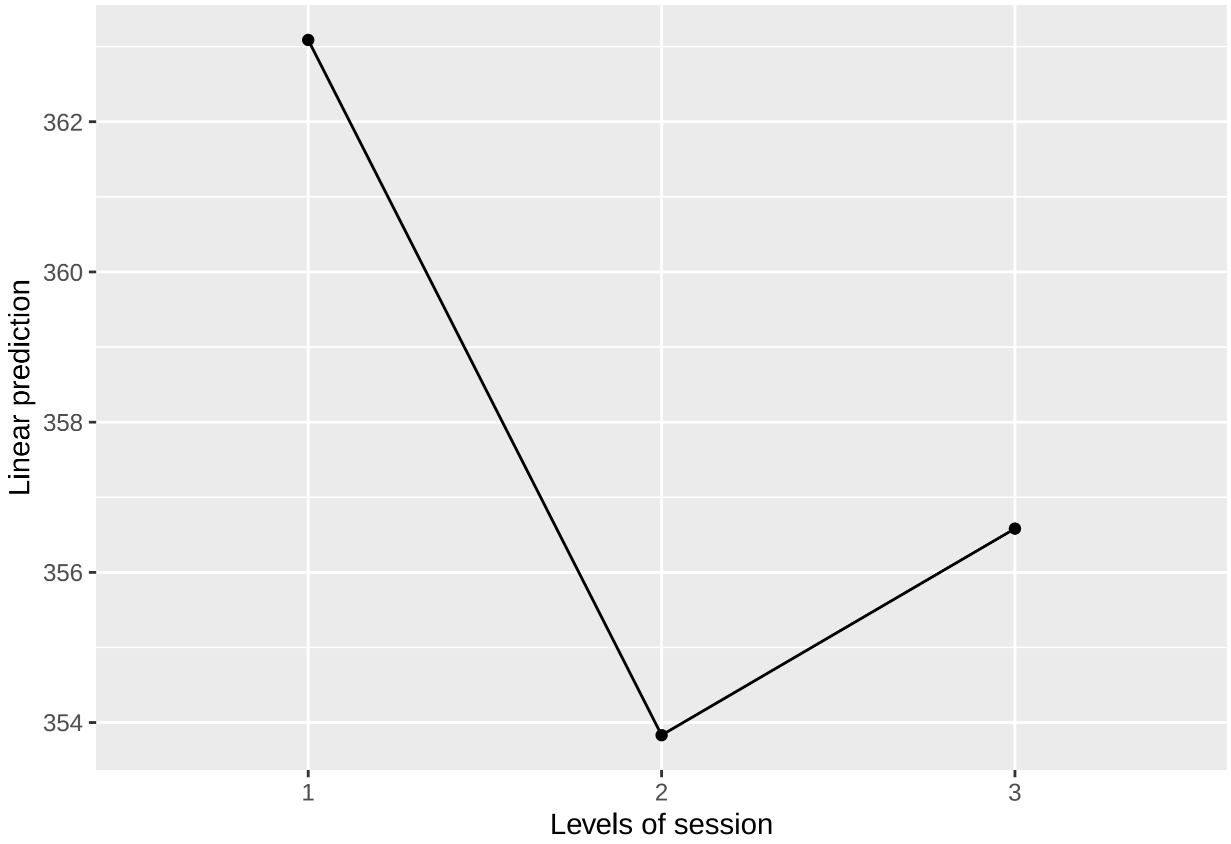


```
## $emmeans
##  session emmean   SE   df lower.CL upper.CL
##  1          363 8.13 29.6      343      384
##  2          354 8.16 30.1      333      374
##  3          357 8.20 30.7      336      377
##
## Results are averaged over the levels of: condition, timePoint, congruency, gender
## Degrees-of-freedom method: satterthwaite
## Confidence level used: 0.95
## Conf-level adjustment: sidak method for 3 estimates
##
## $contrasts
##  contrast estimate   SE  df t.ratio p.value
##  1 - 2        9.26 2.84 428   3.263  0.0034
##  1 - 3        6.51 2.93 427   2.224  0.0684
##  2 - 3       -2.75 2.76 421  -0.998  0.5783
##
## Results are averaged over the levels of: condition, timePoint, congruency, gender
## Degrees-of-freedom method: satterthwaite
## P value adjustment: tukey method for comparing a family of 3 estimates
```

```
##  contrast effect.size    SE  df lower.CL upper.CL
##  (1 - 2)        0.389 0.120 428   0.1532    0.624
##  (1 - 3)        0.273 0.123 427   0.0311    0.515
##  (2 - 3)       -0.116 0.116 421  -0.3431    0.112
##
## Results are averaged over the levels of: condition, timePoint, congruency, gender
## sigma used for effect sizes: 23.82
## Degrees-of-freedom method: inherited from satterthwaite when re-gridding
## Confidence level used: 0.95
```

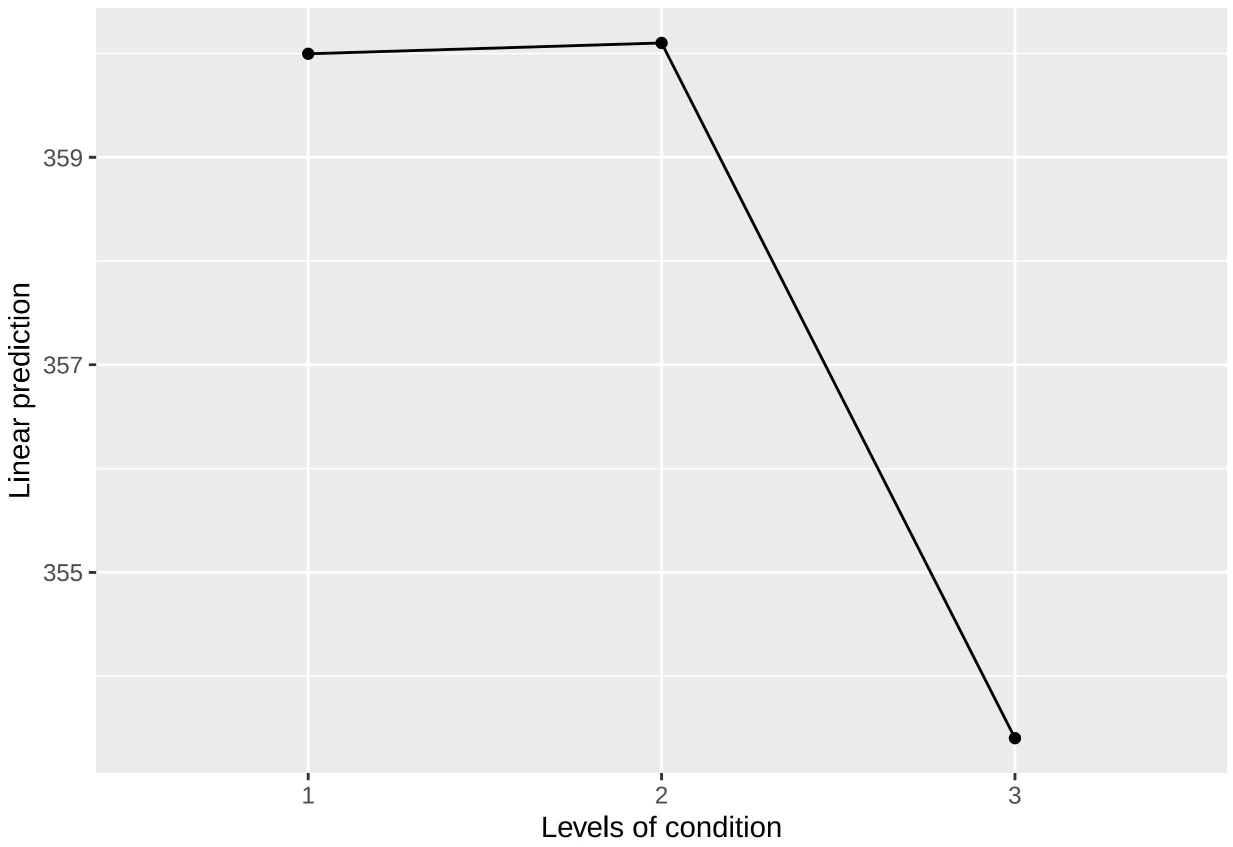


```
## $emmeans
##  condition emmean   SE   df lower.CL upper.CL
##  1            360 8.16 30.1      339      381
##  2            360 8.16 30.1      339      381
##  3            353 8.14 29.8      333      374
##
## Results are averaged over the levels of: session, timePoint, congruency, gender
## Degrees-of-freedom method: satterthwaite
## Confidence level used: 0.95
## Conf-level adjustment: sidak method for 3 estimates
##
## $contrasts
##  contrast estimate   SE  df t.ratio p.value
##  1 - 2      -0.104 2.75 422 -0.038  0.9992
##  1 - 3       6.596 2.80 426  2.355  0.0496
##  2 - 3       6.700 2.76 424  2.424  0.0416
##
## Results are averaged over the levels of: session, timePoint, congruency, gender
## Degrees-of-freedom method: satterthwaite
## P value adjustment: tukey method for comparing a family of 3 estimates

##  contrast effect.size    SE  df lower.CL upper.CL
##  (1 - 2)     -0.00438 0.116 422  -0.2316    0.223
##  (1 - 3)      0.27694 0.118 426   0.0451    0.509
##  (2 - 3)      0.28133 0.116 424   0.0525    0.510
##
## Results are averaged over the levels of: session, timePoint, congruency, gender
## sigma used for effect sizes: 23.82
## Degrees-of-freedom method: inherited from satterthwaite when re-gridding
## Confidence level used: 0.95
```

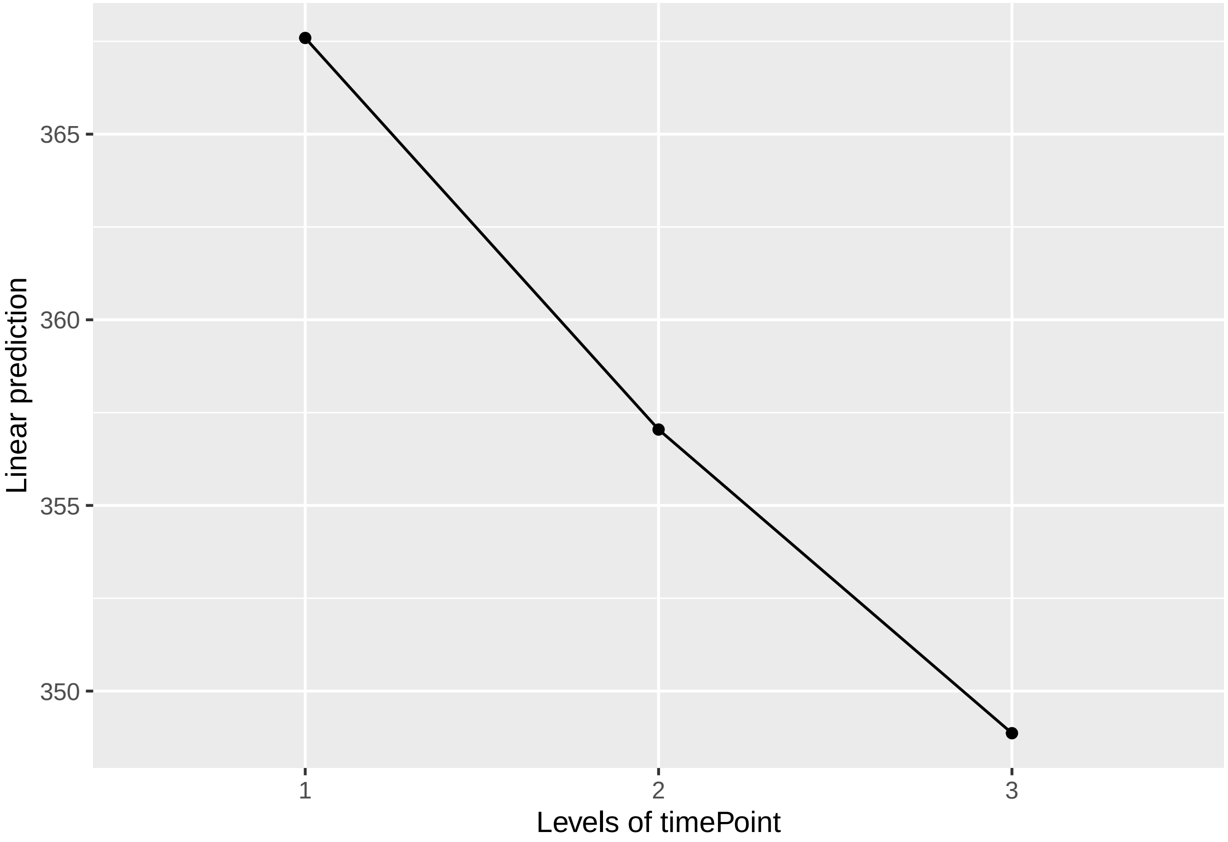


```
## $emmeans
##  timePoint emmean   SE   df lower.CL upper.CL
##  1            368 8.14 29.8      347      388
##  2            357 8.14 29.8      336      378
##  3            349 8.14 29.8      328      369
##
## Results are averaged over the levels of: session, condition, congruency, gender
## Degrees-of-freedom method: satterthwaite
## Confidence level used: 0.95
## Conf-level adjustment: sidak method for 3 estimates
##
## $contrasts
##  contrast estimate   SE  df t.ratio p.value
##  1 - 2       10.55 2.65 420 3.982   0.0002
##  1 - 3       18.73 2.65 420 7.062   <.0001
##  2 - 3        8.18 2.64 420 3.097   0.0059
##
## Results are averaged over the levels of: session, condition, congruency, gender
## Degrees-of-freedom method: satterthwaite
## P value adjustment: tukey method for comparing a family of 3 estimates
```

```
##  contrast effect.size    SE  df lower.CL upper.CL
##  (1 - 2)        0.443 0.112 420    0.222    0.663
##  (1 - 3)        0.786 0.114 420    0.561    1.011
##  (2 - 3)        0.343 0.111 420    0.124    0.563
##
## Results are averaged over the levels of: session, condition, congruency, gender
## sigma used for effect sizes: 23.82
## Degrees-of-freedom method: inherited from satterthwaite when re-gridding
## Confidence level used: 0.95
```

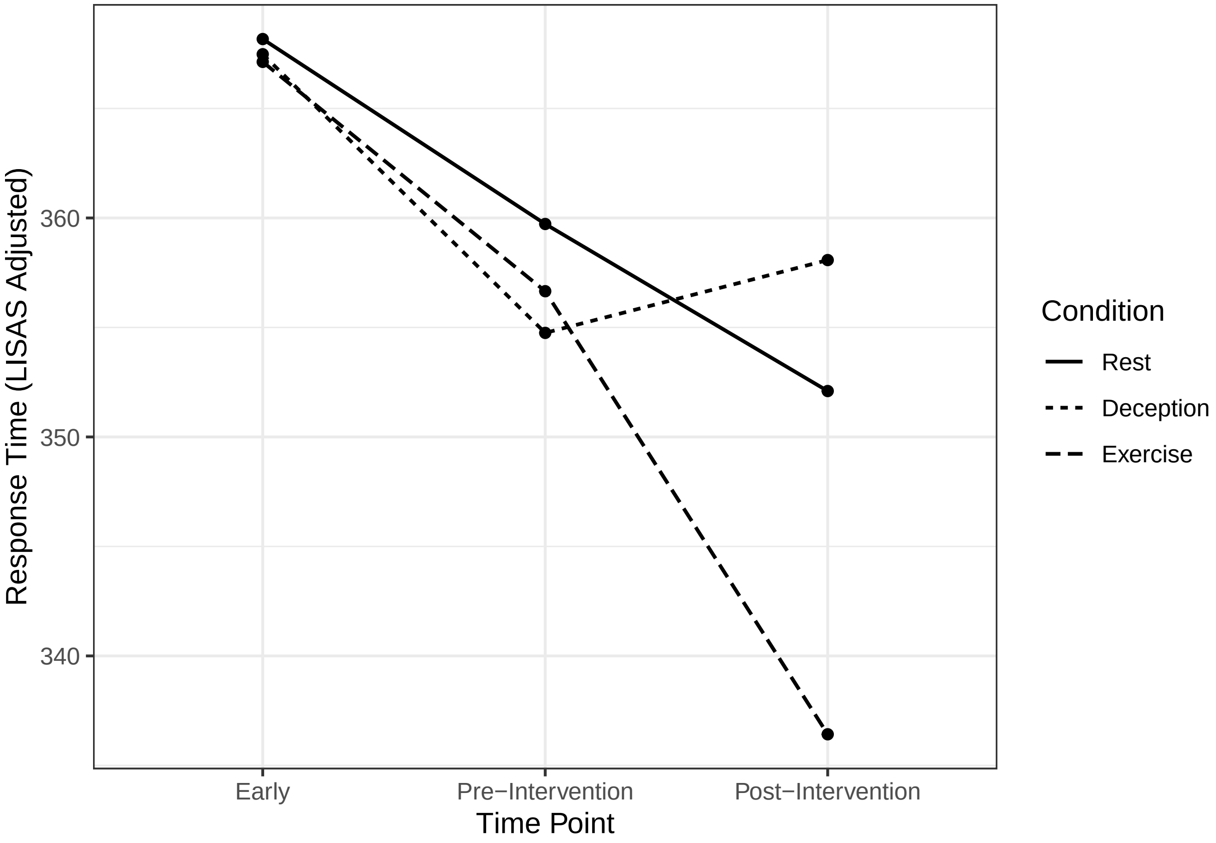


```
## $emmeans
##  condition timePoint emmean   SE   df lower.CL upper.CL
##  1         1            368 8.57 36.5      343      393
##  2         1            367 8.59 37.0      342      393
##  3         1            367 8.57 36.5      342      392
##  1         2            360 8.58 36.7      335      385
##  2         2            355 8.59 37.0      330      380
##  3         2            357 8.54 36.1      332      382
##  1         3            352 8.57 36.6      327      377
##  2         3            358 8.62 37.4      333      383
##  3         3            336 8.54 36.1      311      362
##
## Results are averaged over the levels of: session, congruency, gender
## Degrees-of-freedom method: satterthwaite
## Confidence level used: 0.95
## Conf-level adjustment: sidak method for 9 estimates
##
## $contrasts
##  contrast  estimate   SE  df t.ratio p.value
##  1 1 - 2 1    0.694 4.67 421  0.148  1.0000
##  1 1 - 3 1    1.043 4.66 422  0.224  1.0000
##  1 1 - 1 2    8.445 4.54 420  1.861  0.6408
##  1 1 - 2 2   13.419 4.63 420  2.898  0.0920
##  1 1 - 3 2   11.514 4.60 422  2.504  0.2329
##  1 1 - 1 3   16.073 4.53 420  3.544  0.0129
##  1 1 - 2 3   10.093 4.67 420  2.161  0.4330
##  1 1 - 3 3   31.748 4.62 422  6.865  <.0001
##  2 1 - 3 1    0.349 4.69 421  0.074  1.0000
##  2 1 - 1 2    7.751 4.70 421  1.649  0.7768
##  2 1 - 2 2   12.725 4.67 420  2.724  0.1425
##  2 1 - 3 2   10.821 4.63 421  2.337  0.3225
##  2 1 - 1 3   15.380 4.69 421  3.281  0.0306
```

```
##  2 1 - 2 3    9.399 4.71 420  1.995  0.5477
##  2 1 - 3 3   31.054 4.63 421  6.711  <.0001
##  3 1 - 1 2    7.402 4.68 423  1.580  0.8152
##  3 1 - 2 2   12.376 4.69 421  2.637  0.1747
##  3 1 - 3 2   10.472 4.54 420  2.305  0.3413
##  3 1 - 1 3   15.030 4.67 422  3.215  0.0374
##  3 1 - 2 3    9.050 4.73 421  1.913  0.6054
##  3 1 - 3 3   30.705 4.55 420  6.751  <.0001
##  1 2 - 2 2    4.974 4.64 420  1.072  0.9779
##  1 2 - 3 2    3.069 4.62 422  0.665  0.9992
##  1 2 - 1 3    7.628 4.53 420  1.682  0.7572
##  1 2 - 2 3    1.648 4.68 420  0.352  1.0000
##  1 2 - 3 3   23.303 4.65 423  5.010  <.0001
##  2 2 - 3 2   -1.904 4.64 421 -0.411  1.0000
##  2 2 - 1 3    2.655 4.63 420  0.573  0.9997
##  2 2 - 2 3   -3.326 4.69 420 -0.709  0.9987
##  2 2 - 3 3   18.329 4.65 421  3.940  0.0030
##  3 2 - 1 3    4.559 4.61 422  0.989  0.9867
##  3 2 - 2 3   -1.421 4.67 421 -0.304  1.0000
##  3 2 - 3 3   20.233 4.49 420  4.505  0.0003
##  1 3 - 2 3   -5.980 4.67 420 -1.279  0.9370
##  1 3 - 3 3   15.675 4.64 422  3.380  0.0223
##  2 3 - 3 3   21.655 4.69 421  4.618  0.0002
##
## Results are averaged over the levels of: session, congruency, gender
## Degrees-of-freedom method: satterthwaite
## P value adjustment: tukey method for comparing a family of 9 estimates
```

```
##  contrast    effect.size    SE  df lower.CL upper.CL
##  (1 1 - 2 1)      0.0291 0.196 421 -0.35666    0.415
##  (1 1 - 3 1)      0.0438 0.196 422 -0.34124    0.429
##  (1 1 - 1 2)      0.3546 0.191 420 -0.02057    0.730
##  (1 1 - 2 2)      0.5634 0.195 420  0.17953    0.947
##  (1 1 - 3 2)      0.4835 0.194 422  0.10266    0.864
##  (1 1 - 1 3)      0.6749 0.192 420  0.29801    1.052
##  (1 1 - 2 3)      0.4238 0.197 420  0.03739    0.810
##  (1 1 - 3 3)      1.3331 0.199 422  0.94150    1.725
##  (2 1 - 3 1)      0.0147 0.197 421 -0.37254    0.402
##  (2 1 - 1 2)      0.3255 0.198 421 -0.06305    0.714
##  (2 1 - 2 2)      0.5343 0.197 420  0.14720    0.921
##  (2 1 - 3 2)      0.4544 0.195 421  0.07099    0.838
##  (2 1 - 1 3)      0.6458 0.198 421  0.25659    1.035
##  (2 1 - 2 3)      0.3947 0.198 420  0.00495    0.784
##  (2 1 - 3 3)      1.3039 0.199 421  0.91255    1.695
##  (3 1 - 1 2)      0.3108 0.197 423 -0.07633    0.698
##  (3 1 - 2 2)      0.5196 0.198 421  0.13074    0.909
##  (3 1 - 3 2)      0.4397 0.191 420  0.06366    0.816
##  (3 1 - 1 3)      0.6311 0.197 422  0.24309    1.019
##  (3 1 - 2 3)      0.3800 0.199 421 -0.01132    0.771
##  (3 1 - 3 3)      1.2893 0.196 420  0.90447    1.674
##  (1 2 - 2 2)      0.2088 0.195 420 -0.17439    0.592
##  (1 2 - 3 2)      0.1289 0.194 422 -0.25239    0.510
##  (1 2 - 1 3)      0.3203 0.191 420 -0.05455    0.695
##  (1 2 - 2 3)      0.0692 0.197 420 -0.31714    0.456
##  (1 2 - 3 3)      0.9785 0.198 423  0.58922    1.368
##  (2 2 - 3 2)     -0.0800 0.195 421 -0.46260    0.303
##  (2 2 - 1 3)      0.1115 0.195 420 -0.27113    0.494
##  (2 2 - 2 3)     -0.1396 0.197 420 -0.52690    0.248
##  (2 2 - 3 3)      0.7696 0.197 421  0.38235    1.157
##  (3 2 - 1 3)      0.1914 0.194 422 -0.18911    0.572
##  (3 2 - 2 3)     -0.0597 0.196 421 -0.44542    0.326
```

```
##  (3 2 - 3 3)      0.8496 0.191 420  0.47472    1.224
##  (1 3 - 2 3)     -0.2511 0.196 420 -0.63729    0.135
##  (1 3 - 3 3)      0.6582 0.196 422  0.27293    1.043
##  (2 3 - 3 3)      0.9093 0.199 421  0.51768    1.301
##
## Results are averaged over the levels of: session, congruency, gender
## sigma used for effect sizes: 23.82
## Degrees-of-freedom method: inherited from satterthwaite when re-gridding
## Confidence level used: 0.95
```

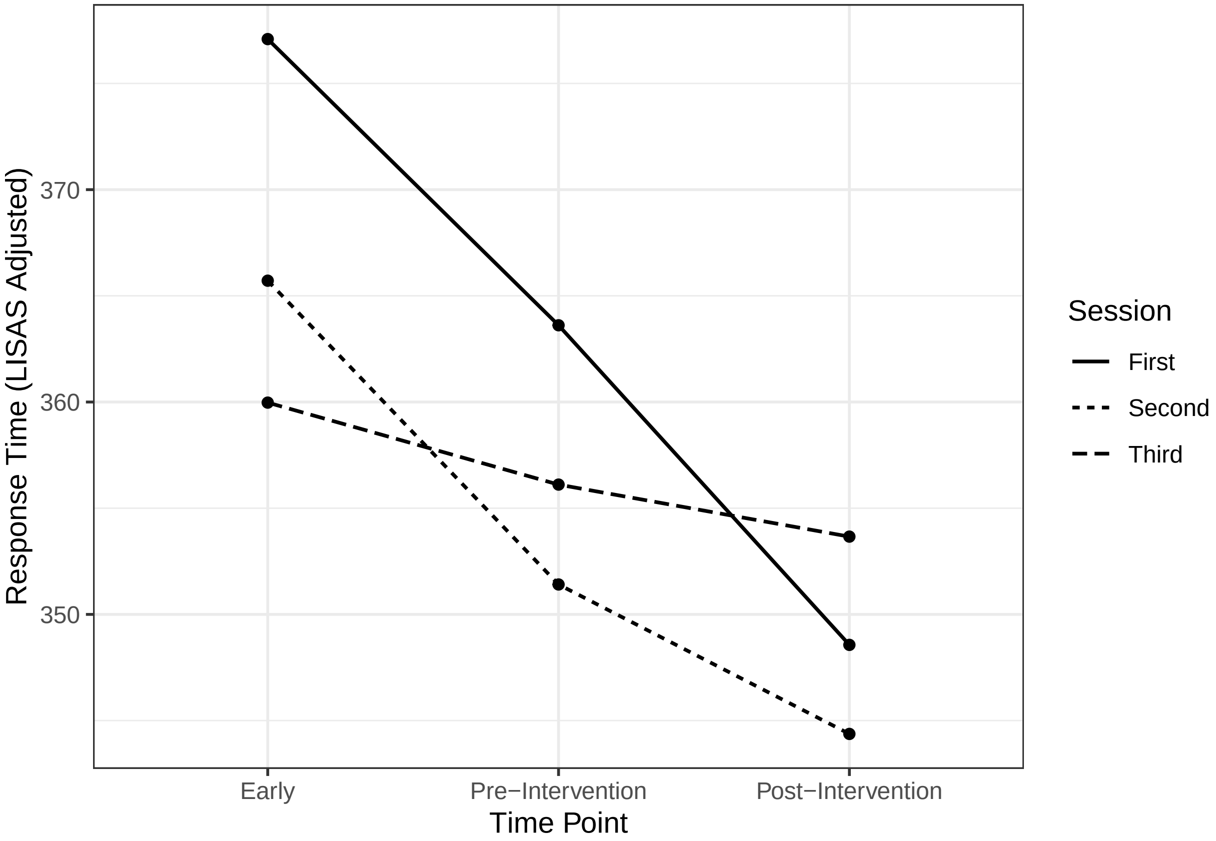
Response Time (LISAS Adjusted)
370
360
350
Early
Pre−Intervention
Post−Intervention
Time Point
Session
First
Second
Third

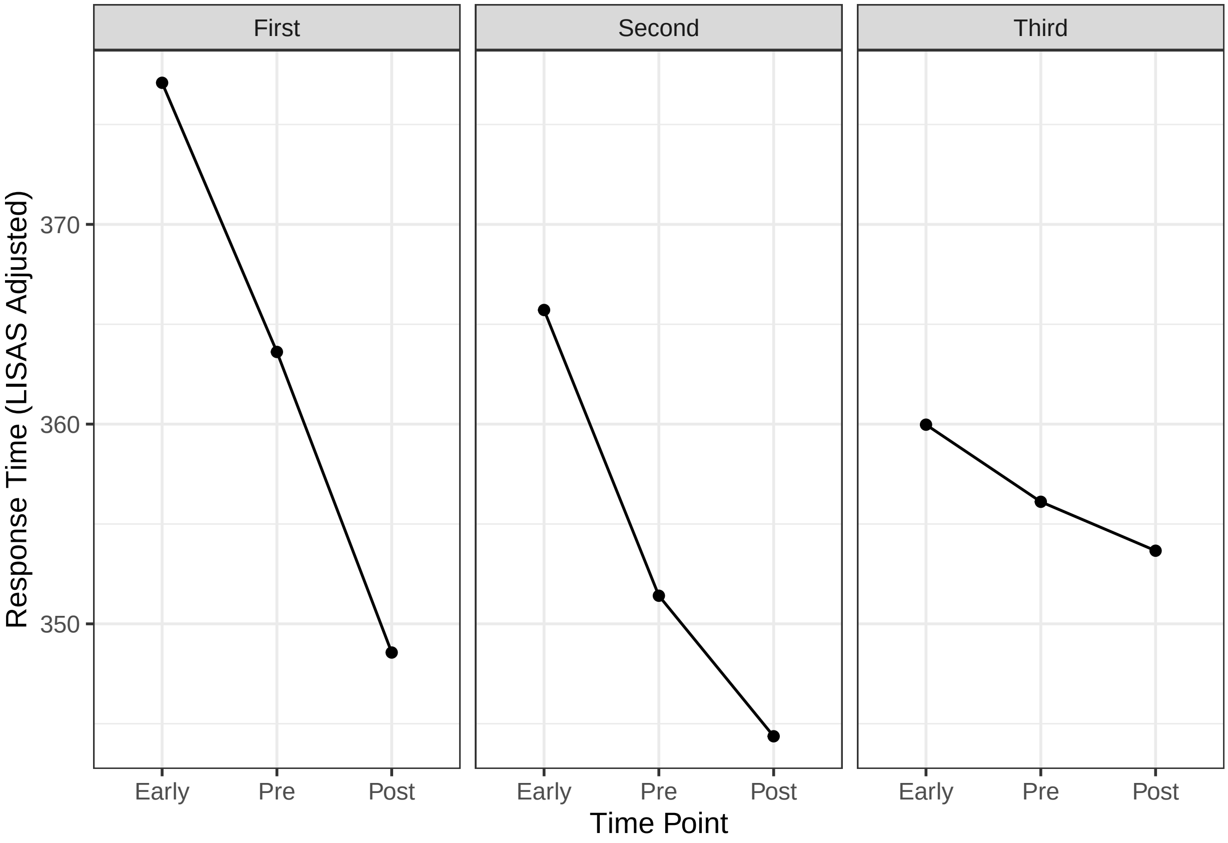


```
## $emmeans
##  session timePoint emmean   SE   df lower.CL upper.CL
##  1       1            377 8.54 36.1      352      402
##  2       1            366 8.56 36.5      341      391
##  3       1            360 8.66 38.2      335      385
##  1       2            364 8.49 35.2      339      389
##  2       2            351 8.58 36.7      326      377
##  3       2            356 8.66 38.1      331      381
##  1       3            349 8.51 35.6      324      374
##  2       3            344 8.56 36.4      319      370
##  3       3            354 8.66 38.2      328      379
##
## Results are averaged over the levels of: condition, congruency, gender
## Degrees-of-freedom method: satterthwaite
## Confidence level used: 0.95
## Conf-level adjustment: sidak method for 9 estimates
##
## $contrasts
##  contrast  estimate   SE  df t.ratio p.value
##  1 1 - 2 1    11.38 4.67 424  2.435  0.2677
##  1 1 - 3 1    17.12 4.85 423  3.532  0.0134
##  1 1 - 1 2    13.48 4.38 420  3.076  0.0565
##  1 1 - 2 2    25.68 4.75 424  5.409  <.0001
##  1 1 - 3 2    20.98 4.83 423  4.343  0.0006
##  1 1 - 1 3    28.53 4.42 420  6.451  <.0001
##  1 1 - 2 3    32.72 4.66 424  7.015  <.0001
##  1 1 - 3 3    23.43 4.85 423  4.827  0.0001
##  2 1 - 3 1     5.74 4.71 420  1.219  0.9521
##  2 1 - 1 2     2.10 4.53 422  0.463  0.9999
##  2 1 - 2 2    14.31 4.54 420  3.154  0.0449
##  2 1 - 3 2     9.60 4.71 420  2.039  0.5169
##  2 1 - 1 3    17.15 4.59 423  3.740  0.0065
##  2 1 - 2 3    21.34 4.53 420  4.716  0.0001
```

```
##  2 1 - 3 3    12.05 4.71 420  2.558  0.2077
##  3 1 - 1 2    -3.64 4.71 422 -0.774  0.9975
##  3 1 - 2 2     8.56 4.71 420  1.817  0.6707
##  3 1 - 3 2     3.86 4.82 420  0.800  0.9968
##  3 1 - 1 3    11.41 4.76 422  2.399  0.2873
##  3 1 - 2 3    15.60 4.71 420  3.312  0.0277
##  3 1 - 3 3     6.31 4.82 420  1.309  0.9286
##  1 2 - 2 2    12.21 4.59 423  2.660  0.1655
##  1 2 - 3 2     7.50 4.70 422  1.598  0.8058
##  1 2 - 1 3    15.05 4.35 420  3.457  0.0173
##  1 2 - 2 3    19.24 4.53 422  4.249  0.0009
##  1 2 - 3 3     9.95 4.71 422  2.112  0.4663
##  2 2 - 3 2    -4.70 4.72 420 -0.997  0.9860
##  2 2 - 1 3     2.84 4.64 423  0.612  0.9995
##  2 2 - 2 3     7.04 4.54 420  1.551  0.8306
##  2 2 - 3 3    -2.25 4.71 420 -0.478  0.9999
##  3 2 - 1 3     7.55 4.74 422  1.591  0.8095
##  3 2 - 2 3    11.74 4.71 420  2.493  0.2383
##  3 2 - 3 3     2.45 4.82 420  0.508  0.9999
##  1 3 - 2 3     4.19 4.58 422  0.915  0.9921
##  1 3 - 3 3    -5.10 4.76 422 -1.071  0.9781
##  2 3 - 3 3    -9.29 4.71 420 -1.972  0.5640
##
## Results are averaged over the levels of: condition, congruency, gender
## Degrees-of-freedom method: satterthwaite
## P value adjustment: tukey method for comparing a family of 9 estimates

##  contrast    effect.size    SE  df lower.CL  upper.CL
##  (1 1 - 2 1)      0.4777 0.197 424   0.0908  0.864479
##  (1 1 - 3 1)      0.7187 0.205 423   0.3160  1.121429
##  (1 1 - 1 2)      0.5658 0.185 420   0.2023  0.929338
##  (1 1 - 2 2)      1.0783 0.203 424   0.6802  1.476521
##  (1 1 - 3 2)      0.8808 0.205 423   0.4780  1.283616
##  (1 1 - 1 3)      1.1978 0.190 420   0.8244  1.571083
##  (1 1 - 2 3)      1.3738 0.201 424   0.9785  1.769123
##  (1 1 - 3 3)      0.9837 0.206 423   0.5780  1.389407
##  (2 1 - 3 1)      0.2411 0.198 420  -0.1480  0.630116
##  (2 1 - 1 2)      0.0882 0.190 422  -0.2861  0.462372
##  (2 1 - 2 2)      0.6007 0.191 420   0.2243  0.977065
##  (2 1 - 3 2)      0.4032 0.198 420   0.0136  0.792720
##  (2 1 - 1 3)      0.7201 0.194 423   0.3387  1.101509
##  (2 1 - 2 3)      0.8961 0.192 420   0.5180  1.274275
##  (2 1 - 3 3)      0.5060 0.199 420   0.1158  0.896244
##  (3 1 - 1 2)     -0.1529 0.198 422  -0.5415  0.235655
##  (3 1 - 2 2)      0.3596 0.198 420  -0.0301  0.749300
##  (3 1 - 3 2)      0.1621 0.203 420  -0.2361  0.560319
##  (3 1 - 1 3)      0.4790 0.200 422   0.0852  0.872817
##  (3 1 - 2 3)      0.6551 0.199 420   0.2639  1.046226
##  (3 1 - 3 3)      0.2650 0.203 420  -0.1334  0.663397
##  (1 2 - 2 2)      0.5125 0.193 423   0.1323  0.892716
##  (1 2 - 3 2)      0.3150 0.197 422  -0.0731  0.703094
##  (1 2 - 1 3)      0.6319 0.184 420   0.2702  0.993674
##  (1 2 - 2 3)      0.8080 0.192 422   0.4305  1.185463
##  (1 2 - 3 3)      0.4179 0.198 422   0.0281  0.807723
##  (2 2 - 3 2)     -0.1975 0.198 420  -0.5870  0.191933
##  (2 2 - 1 3)      0.1194 0.195 423  -0.2640  0.502771
##  (2 2 - 2 3)      0.2954 0.191 420  -0.0796  0.670440
##  (2 2 - 3 3)     -0.0946 0.198 420  -0.4836  0.294278
##  (3 2 - 1 3)      0.3169 0.199 422  -0.0752  0.709028
##  (3 2 - 2 3)      0.4930 0.198 420   0.1029  0.882981
##  (3 2 - 3 3)      0.1029 0.203 420  -0.2953  0.501045
```

```
##  (1 3 - 2 3)      0.1760 0.192 422  -0.2021  0.554219
##  (1 3 - 3 3)     -0.2141 0.200 422  -0.6073  0.179192
##  (2 3 - 3 3)     -0.3901 0.198 420  -0.7798 -0.000374
##
## Results are averaged over the levels of: condition, congruency, gender
## sigma used for effect sizes: 23.82
## Degrees-of-freedom method: inherited from satterthwaite when re-gridding
## Confidence level used: 0.95
```

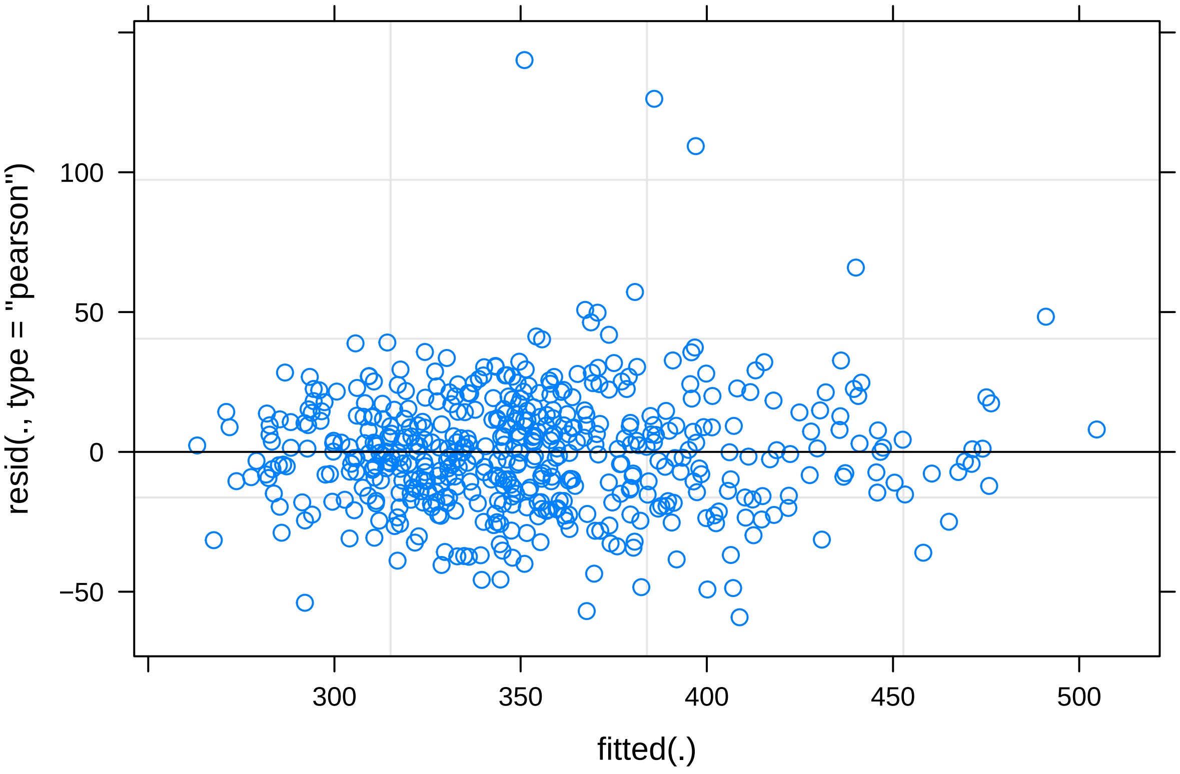
resid(., type = "pearson")
fitted(.)
100
50
0
−50
300
350
400
450
500

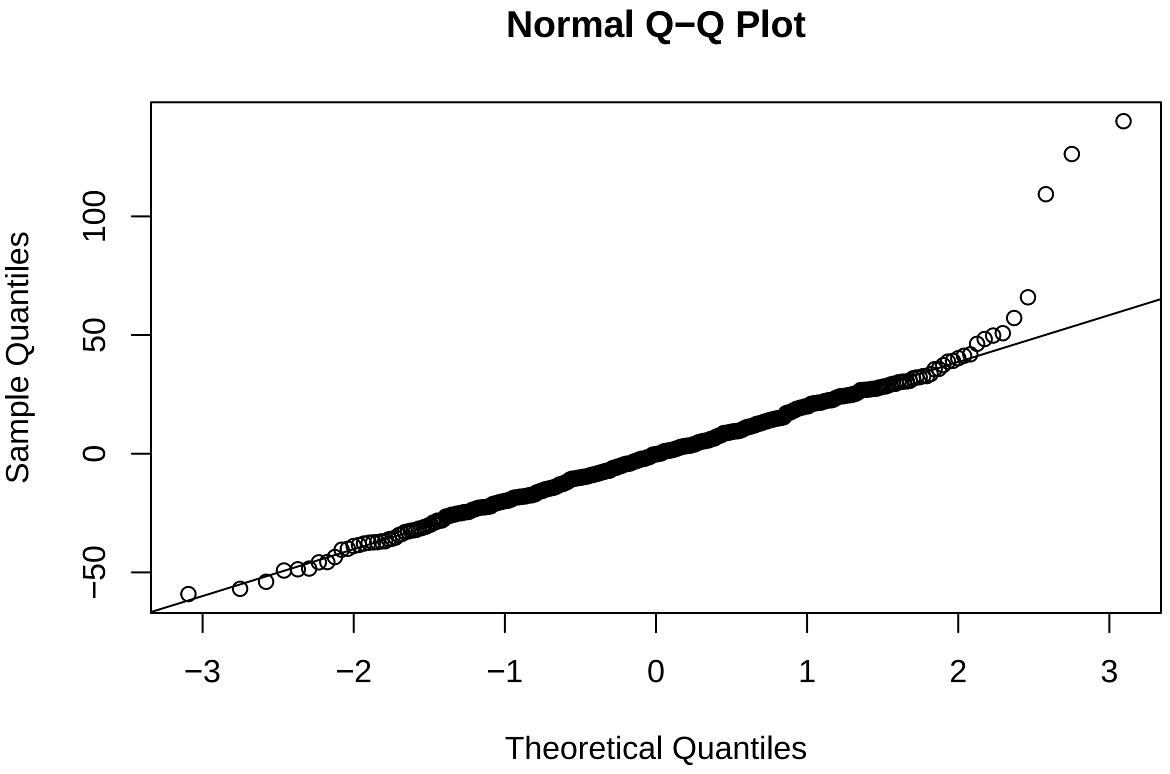
Normal Q−Q Plot
Sample Quantiles
Theoretical Quantiles
100
50
0
−50
−3
−2
−1
0
1
2
3

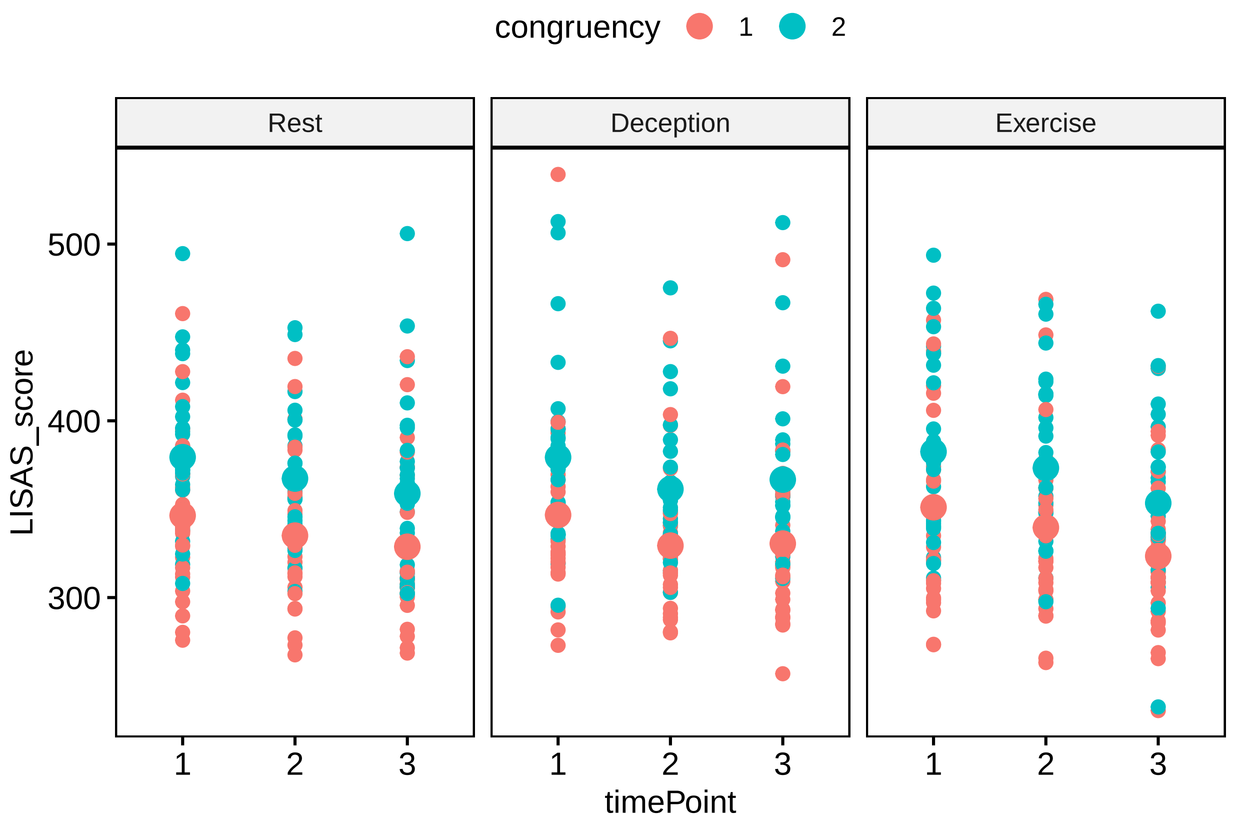
congruency
1
2
Rest
Deception
Exercise
LISAS_score
500
400
300
1
2
3
timePoint

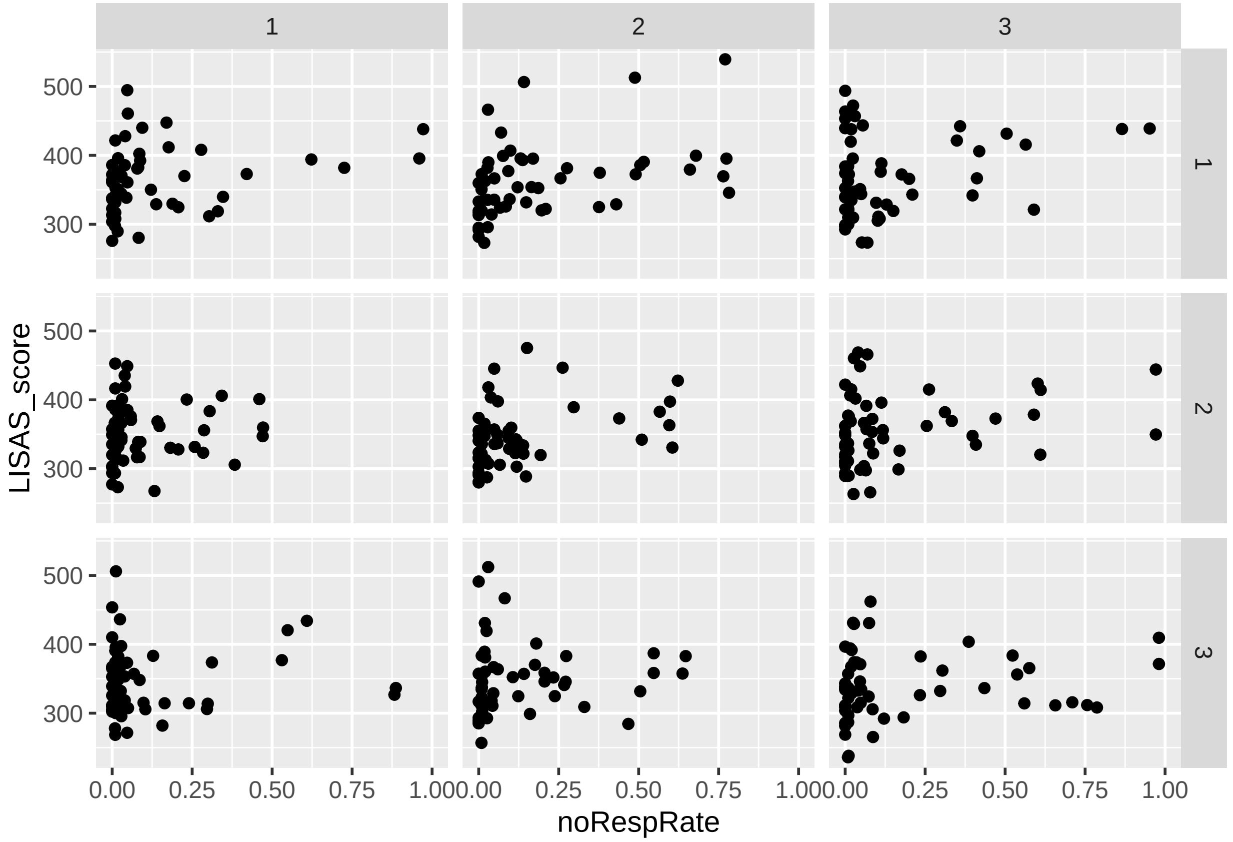
1
2
3
LISAS_score
500
400
300
0.00
0.25
0.50
0.75
1.00
noRespRate

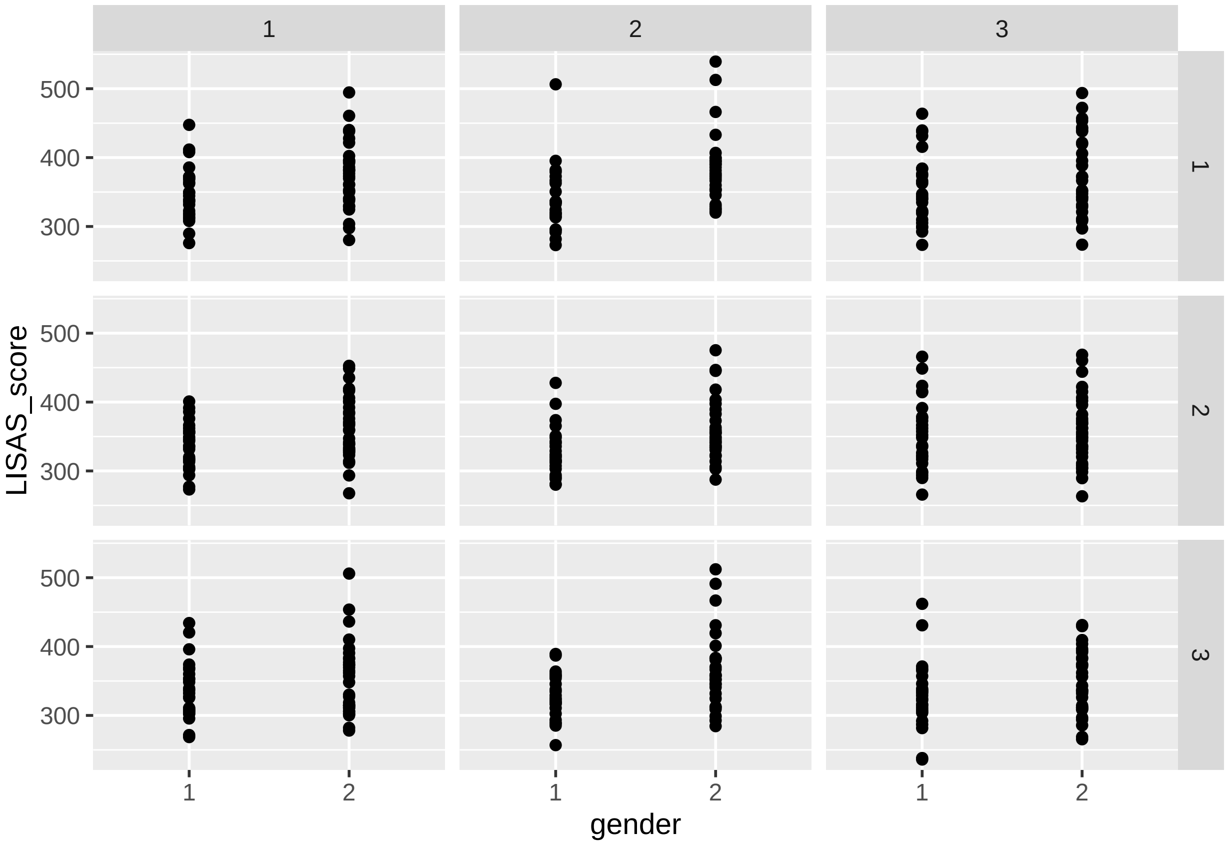
1
2
3
500
400
300
LISAS_score
1
2
3
1
2
gender

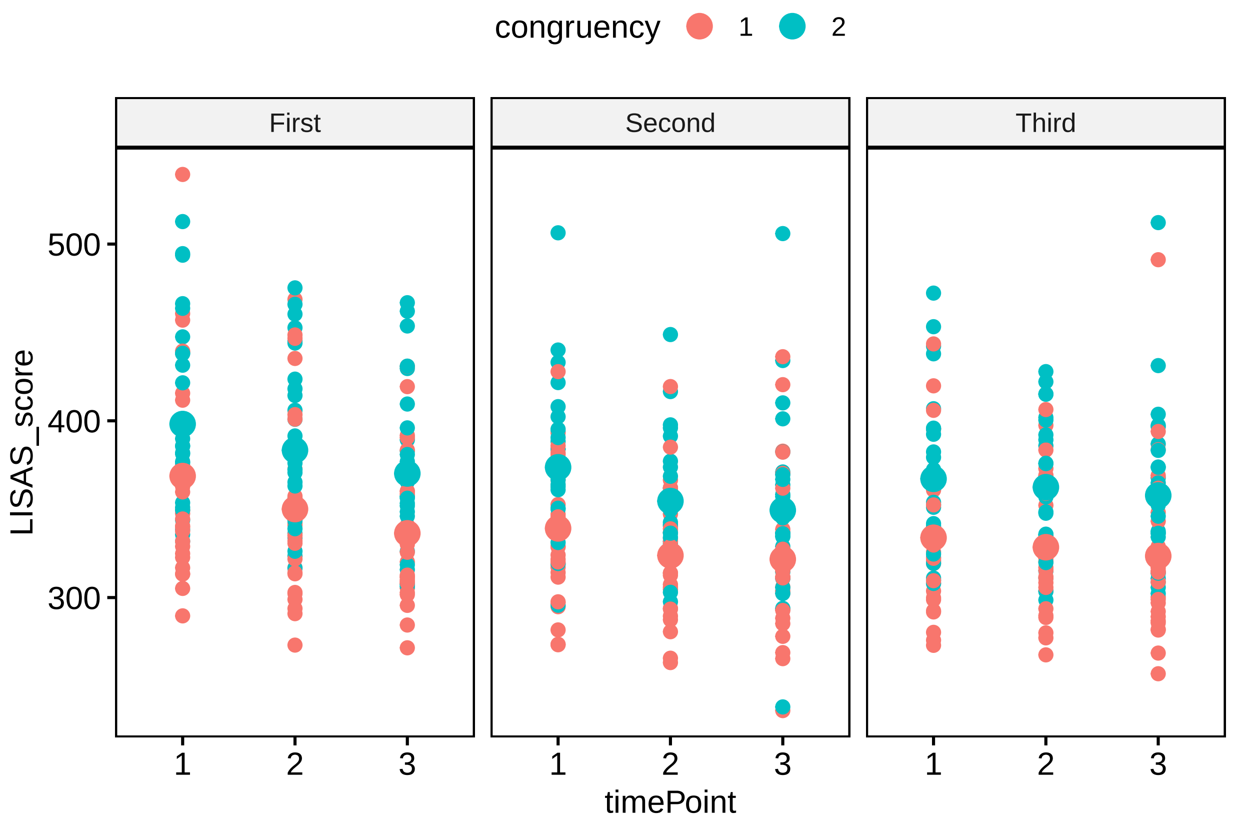
congruency
1
2
First
Second
Third
500
400
300
LISAS_score
1
2
3
timePoint

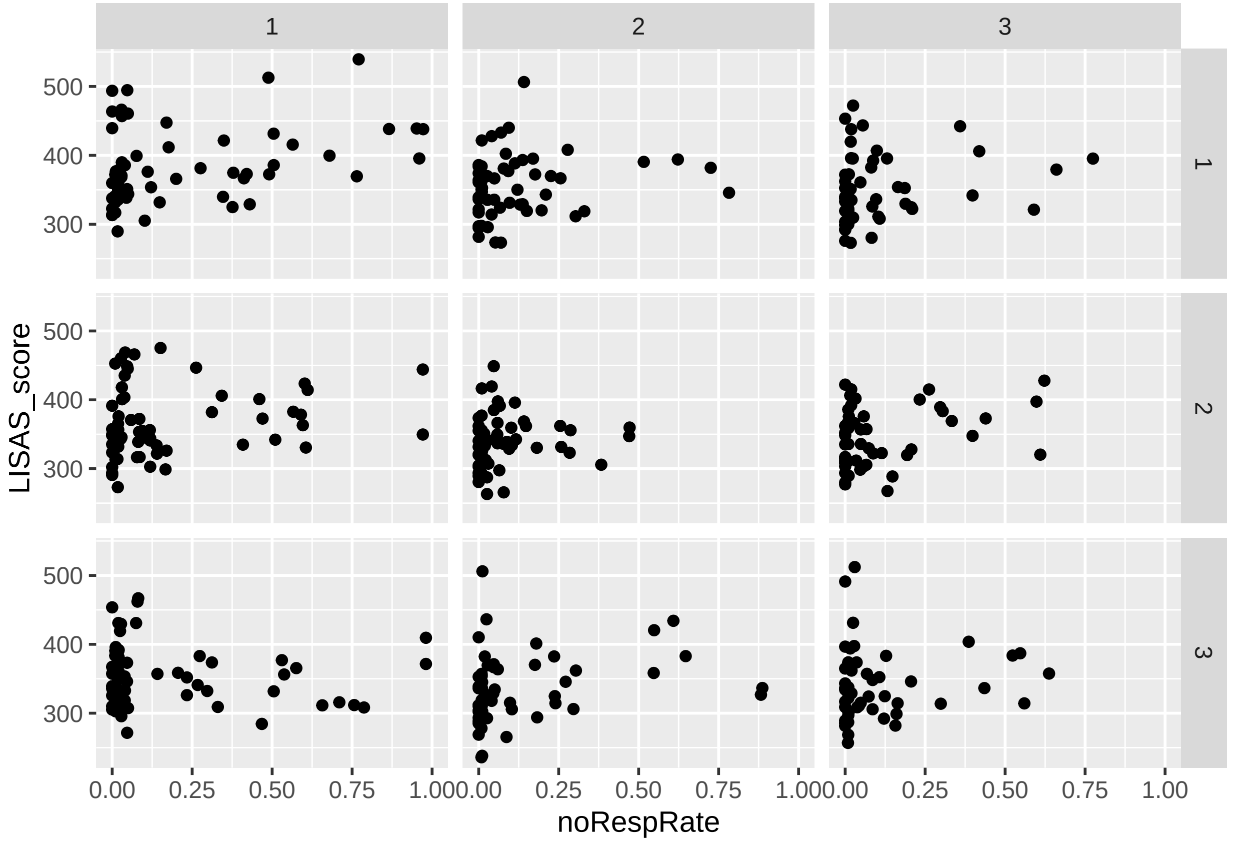
1
2
3
500
400
300
LISAS_score
0.00
0.25
0.50
0.75
1.00
noRespRate

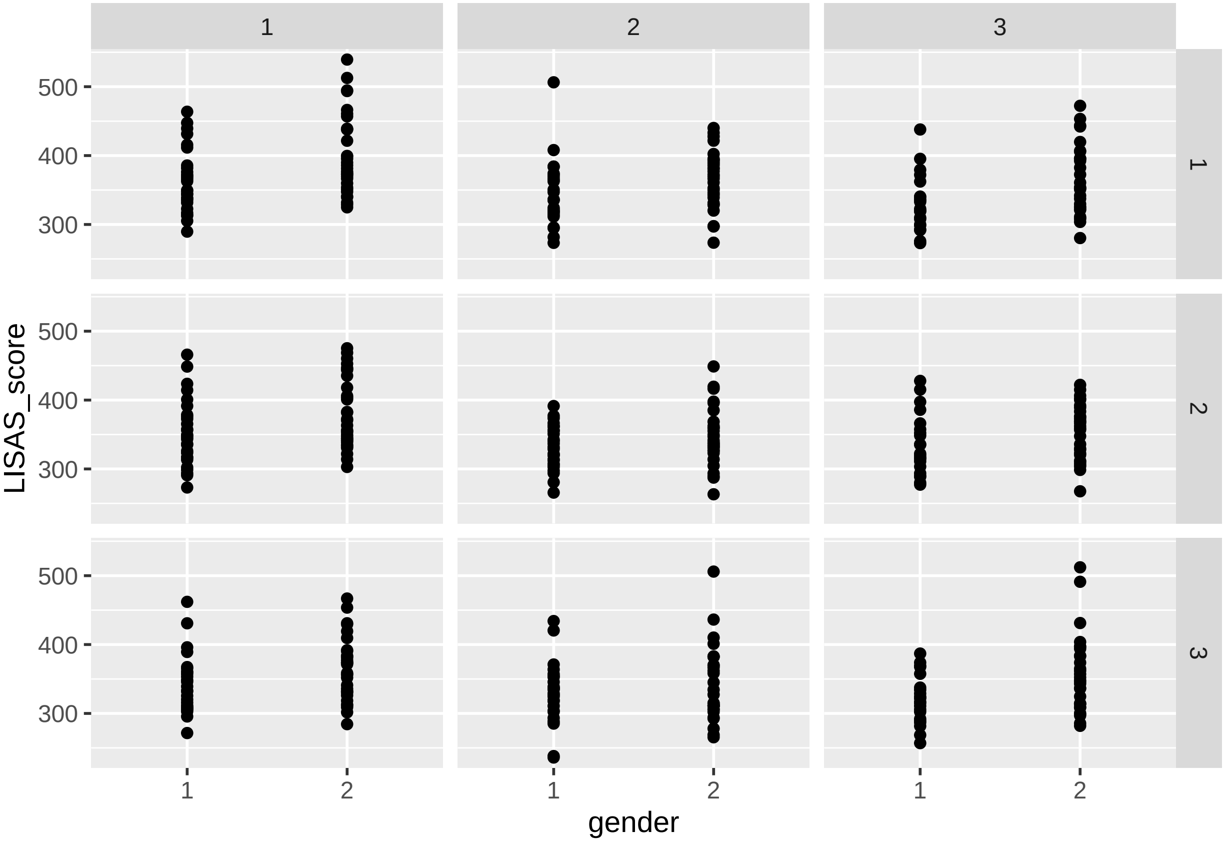
1
2
3
1
2
3
500
400
300
LISAS_score
1
2
gender
