# Supplementary material for: Moderate aerobic exercise, but not anticipation of exercise, improves cognitive control
Source: PLoS One. 2020 Nov 13;15(11):e0242270. doi: 10.1371/journal.pone.0242270 (PMC7665798; doi:10.1371/journal.pone.0242270)
Supplement: Attachment — Submitted filename: PLOSONE_Rebuttal_Letter_Rev_2.docx [file pone.0242270.s003.docx]

*Rebuttal Letter*

PONE-D-20-21829
Moderate aerobic exercise, but not anticipation of exercise, improves cognitive control
PLOS ONE

Dear Dr. Wanner,

Thank you for the opportunity to revise our manuscript as well as the thoughtful comments and suggestions. In our revised manuscript, we have addressed your additional comments with our responses detailed on the next page. Alterations to the manuscript addressing your comments are highlighted in yellow.

Thank you again for your suggestions, we believe that they significantly improve the clarity of this manuscript.

Best regards,

Max Bergelt

Additional Editor Comments:

1) To define the meaning of the abbreviation SMD and explain how the standardized mean differences were calculated. Please also indicate the threshold values used to classify the effects sizes based on the SMD calculation.

*>We have added the meaning of SMD and explained how it was calculated in section* ‘2.5 Statistical analysis’*. We have also added thresholds for small, medium, and large effects in order to clarify interpretation.*

2) To present the heart rate data as absolute values and indicate the percentage of maximum heart rate for specific time points, particularly after the exercise.

*>We now present both the absolute heart rates at each time point, as well as percentage of maximum heart rate. The absolute heart rate is presented in* **Table 1***. The %HR max during and after the intervention are presented in the text of* ‘3.2 Exercise characteristics’*.*
